# Architecture of an Electrical Equivalence Pyranometer with Temperature Difference Analog Control

**DOI:** 10.3390/s22218137

**Published:** 2022-10-24

**Authors:** Evandson Claude Seabra Dantas, José Taunaí Dantas Segundo, Sebastian Yuri Cavalcanti Catunda, Diomadson Rodrigues Belfort, Raimundo Carlos Silvérios Freire, Paulo Fernandes da Silva Júnior

**Affiliations:** 1 Graduate Program in Electrical and Computer Engineering, Technology Center, Federal University of Rio Grande do Norte, Natal 59078-970, RN, Brazil; 2 Graduate Program in Electrical Engineering, Department of Electrical Engineering, Federal University of Campina Grande, Campina Grande 58429-900, PB, Brazil; 3 Department of Electrical Engineering, State University of Maranhão, Balsas 65800-000, MA, Brazil

**Keywords:** analog pyranometer, analog radiometer, electrical equivalence, electrical substitution, constant temperature difference, Wheatstone bridge

## Abstract

In this paper, an architecture of an electrical equivalence pyranometer with analog control of the temperature difference is presented. The classical electrical equivalence pyranometer employs a Wheatstone bridge with a feedback amplifier to keep the sensor operating at a constant temperature to estimate the incident radiation through the sensor thermal balance employing the electrical equivalence principal. However, this architecture presents limitations under ambient temperature variation, such as sensitivity variation. To overcome those limitations, we propose an architecture that controls the temperature difference between the sensor and ambient via an analog compensating circuit. Analytical results show an improvement near five times in sensitivity over the ambient temperature span and 76.3% increase of useful output voltage. A prototype was developed and validated with a commercial pyranometer, showing a high agreement on the measurement results. It is verified that the use of temperature difference, rather than constant temperature, significantly reduces the effect of ambient temperature variation.

## 1. Introduction

Pyranometers are devices used to measure solar radiation that are composed of direct and diffuse incident radiation [1]. It is used, for example, to measure the energy transfer rate per unit area to produce the Global Horizontal Irradiation (GHI), which, in turn, is used to determine the energy potential for photovoltaic systems in a region [2].

According to the ISO Norm 9060:2018 [3], there are two pyranometer types: (i) the silicon-cell pyranometer, which provides a fast time response but with limited spectral range; and (ii) the thermopile pyranometer, which provides a high and flat spectral range but with a slow time response. Alternatively, there is a class of pyranometers based on the Electrical Equivalence Principle (EEP) or Electrical Substitution Principle (ESP). The EEP uses a feedback architecture to self-balance the sensor heat dissipation with the sensor incoming power, for which the latter is composed of the electrical power and the incident radiation power. The incident radiation can, then, be estimated through the thermal balance equation [4,5,6,7].

The majority of pyranometers based on the EEP operate at a constant temperature, which has the main benefit of reducing the response time [8,9]. The classical analog architecture is composed of a two-analog Wheatstone Bridge in a differential voltage configuration, employing two sensors [6]. One sensor is painted in black and is used mainly for sensing the incident radiation, while the other one is painted in white and serves to compensate the influence of the ambient temperature. Similarly, other constant temperature architectures, like the PWM [10] and the Sigma-Delta [7,11], also employ two sensors.

The ambient temperature directly affects the sensor thermal balance, which, for the architecture with the sensor operating at a constant temperature, has an important effect on the sensitivity of the system, among other parameters [1]. In this paper, we propose an analog pyranometer architecture based on the EEP that operates with a controlled temperature difference, which has the main advantage of mitigating the influence of ambient temperature variation of the system performance. The analysis performed show five times sensibility improvement, reduction of the ambient temperature influence, and 76.3% increase in the output voltage useful span.

The remainder of this paper is organized as follows: Section 2 presents the principle of electrical equivalence applied to the classical architecture of the Wheatstone bridge. Limitations of the bridge in relation to the variation of the ambient temperature are demonstrated. A method for changing the sensor operation and the necessary modifications of the classical architecture to transform them into the proposed architecture is also proposed. In Section 3, an analytical analysis of the proposed architecture is carried out, followed by a validation by computer simulation. In Section 4, general considerations about the work developed are presented, as well as the main contributions of the work. Section 5 contains the final conclusions and, in Section 6, patents deposited from this research are presented.

## 2. Material and Methods

The classical pyranometers based on EEP control the temperature of a thermoresistive sensor through the Joule effect to keep it constant. As consequence, the incident radiation can be estimated by the thermal balance, while the feedback control reduces the system response time.

### 2.1. State of the Art

The electrical equivalence principle is historically used for measure incident radiation and fluids speeds. In recent years, there has been a growing trend to replace analog architecture with digital for the greater ease of correction of ambient temperature variation. In addition, many works have contributed to the study of uncertainties and the improvement of sensitivity. Table 1 summarizes the State of the Art in the last 6 years.

### 2.2. Thermal Balance in Electrical Equivalence Pyranometers

A thermoresistive sensor varies its resistance with the temperature, and can present either a negative or a positive temperature coefficient, called NTC and PTC, respectively. NTC sensors have a nonlinear resistance–temperature relationship but generally a much higher sensitivity than PTC, and, for this reason, they are chosen. The NTC sensor resistance can be approximated by the β-model equation as [17,18]:

(1) $$R_{s}=R_{25}\cdot \exp\left(\frac{\beta }{T_{s}+273.15}-\frac{\beta }{298.15}\right),$$

where $R_{25}$ [Ω] represents the electrical resistance at 25 ${}^{\circ }$C, β [K] is the intrinsic thermistor temperature, and $T_{s}$ [${}^{\circ }$C] is the sensor temperature.

The sensor thermal balance equation relates its temperature with the sensor incoming and lost power, and can be given by [6,7,14,19,20,21]:

(2) $$P_{e}+P_{h}=G_{th}\cdot \left(T_{s}-T_{a}\right)+C_{th}\frac{dT_{s}}{dt},$$

where $P_{e}$ [W] represents the electrical power, $P_{h}$ [W] represents the absorbed radiation power, $G_{th}$ [W· ${}^{\circ }$C${}^{-1}$] is the thermal dissipation factor, $C_{th}$ [J· ${}^{\circ }$C${}^{-1}$] the thermal capacity, $T_{s}$ [${}^{\circ }$C] the sensor temperature, and $T_{a}$ [${}^{\circ }$C] the ambient temperature.

**Figure 1:**
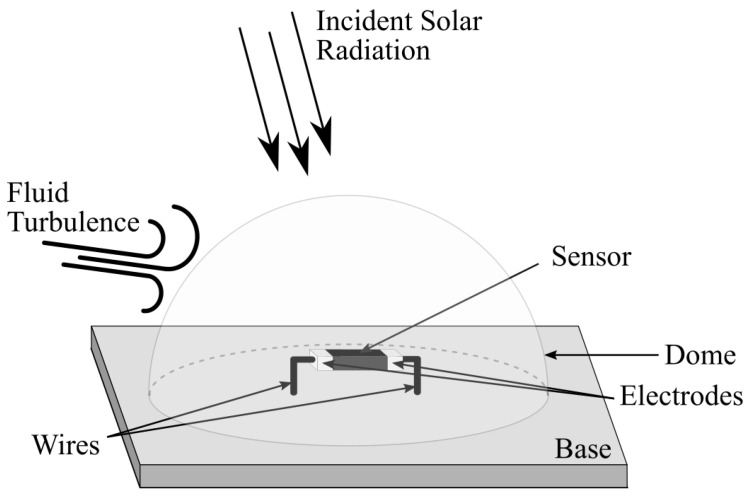
The sensor module used in Electrical Equivalence Pyranometers.

The thermal dissipation factor $G_{th}$ is nonlinearly dependent on the fluid dynamics, and to avoid the influence of the latter, a transparent dome is used. Hence, the thermal dissipation factor can be approximated by a constant [22,23]. The sensor module, composed of a transparent dome, the sensor, and a supporting base, is illustrated in Figure 1.

The power absorbed by sensor due to the incident radiation, $P_{h}$, expressed as [24,25]:

(3) $$P_{h}=\alpha \cdot H,$$

where *H* is the incident radiation power per unit area [W/m${}^{2}$], and α can be viewed as a calibration constant [m${}^{2}$], which is composed of the dome transmittance, sensor absorption coefficient, and exposed sensor area to the incident radiation.

In steady-state, (2) can be written as:

(4) $$\frac{V_{s}^{2}}{R_{s}}+\alpha \cdot H=G_{th}\cdot \left(T_{s}-T_{a}\right),$$

where $V_{s}$ represents the voltage across the sensor and $R_{s}$ the sensor resistance.

### 2.3. Constant Temperature Wheatstone Bridge Architecture

**Figure 2:**
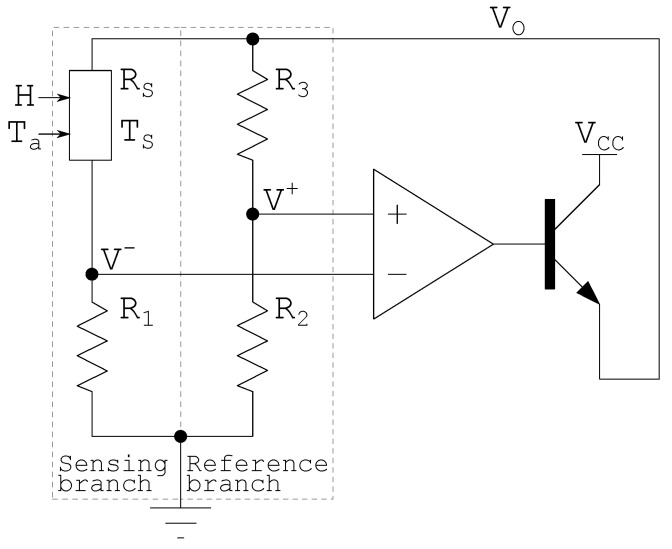
CT Wheatstone bridge schematic using NTC adapted from [6,26,27].

The Constant Temperature (CT) Wheatstone bridge architecture, shown in Figure 2, is responsible for controlling the electrical power delivered to the sensor to keep it at a constant temperature [6,15]. In steady-state, the difference between the sensor heat dissipation and electrical power is equivalent to its absorbed power, and *H* can be estimated from (4).

As shown in Figure 2, the Wheatstone bridge branches are classified as: (i) sensing branch and (ii) the reference branch. As the name suggests, the sensing branch contains a sensor that heats up with electrical energy and incident radiation. The reference branch is used as a reference for the sensor temperature. An operational amplifier (OpAmp) is used to provide a high negative feedback gain making the uncertainties associated with measurements parameters negligible [28]. It may also include a bipolar transistor to provide sufficient current gain to drive the bridge [8,9,29].

Any variation in the incident radiation power tends to change the temperature and resistance of the sensor, and consequently to unbalance the bridge. However, this variation is sensed by the OpAmp, which, due to its high open-loop gain, increases or decreases the electrical output power delivered to the bridge to bring it back to equilibrium, keeping the sensor temperature and resistance constant [14,27].

From Figure 2, neglecting the effect of the OpAmp input voltage offset, in the thermal balance steady-state, the following can be written:

(5) $$\left(V_{o}-V^{-}\right)\cdot \frac{V^{-}}{R_{1}}+\alpha \cdot H=G_{th}\cdot \left(T_{s}-T_{a}\right),$$

where

(6) $$V^{-}=V_{o}\cdot \frac{R_{1}}{R_{1}+R_{s}},$$

and

(7) $$V^{+}=V_{o}\cdot \frac{R_{2}}{R_{2}+R_{3}}.$$

Considering that, in steady state, the voltages $V^{-}$ and $V^{+}$ are approximately equal, it becomes possible to determine the sensor resistance $R_{s}$ that is greater than zero, as a ratio of the resistances of the Wheatstone bridge:

(8) $$R_{s}\approx \frac{R_{1}\cdot R_{3}}{R_{2}},$$

and this relationship is used to implement our proposed architecture.

Recombining (5) and (6), the OpAmp output voltage can be found as:

(9) $$V_{o}=\left(R_{1}+R_{s}\right)\cdot \sqrt{\frac{G_{th}\cdot \left(T_{s}-T_{a}\right)-\alpha \cdot H}{R_{s}}},$$

in which $V_{o}$ is used to calculate the output voltages needed to keep the sensor warm in any condition of ambient temperature and incident radiation.

### 2.4. Constant Temperature Difference Wheatstone Bridge Architecture

The CT architecture features a high feedback gain in the analog domain, which results in a rapid compensation of the electrical power delivered to the sensor. This compensation is directly influenced by the ambient temperature variation that drives the architecture to increase or decrease the output power influencing directly the incident radiation estimation. To preserve the architecture advantage and mitigate the effect caused by ambient temperature variation, it is proposed to use a constant temperature difference instead of constant temperature in Wheatstone bridge architecture. The proposed architecture is called the Constant Temperature Difference (CTD) Wheatstone bridge.

The proposed CTD Wheatstone bridge architecture minimizes the problem of dependence of the output voltage range on the ambient temperature presented by the classical architecture, controlling the sensor temperature so that it is different from the ambient temperature, i.e., $T_{s}-T_{a}$, is constant. Rewriting (9) using $\Delta T=T_{s}-T_{a}$ gives:

(10) $$V_{o}=\left(R_{1}+R_{s}\right)\cdot \sqrt{\frac{G_{th}\cdot \Delta T-\alpha \cdot H}{R_{s}}}$$

with

(11) $$R_{s}=f\left(T_{a}+\Delta T\right).$$

The best value of ΔT in (10) is the one that ensures no output signal saturation for the maximum incident radiation and maximizes the architecture sensitivity. For maximizing the sensitivity, it is desirable for the output voltage ($V_{o}$) to be zero for the maximum incident radiation. Hence, ΔT can be defined as:

(12) $$\Delta T=\frac{\alpha \cdot \max\left(H\right)}{G_{th}}.$$

Keeping the sensor at ΔT above the ambient temperature requires some modifications in the Wheatstone bridge reference branch. The main change is the addition of a second sensor used to sense the ambient temperature.

**Figure 3:**
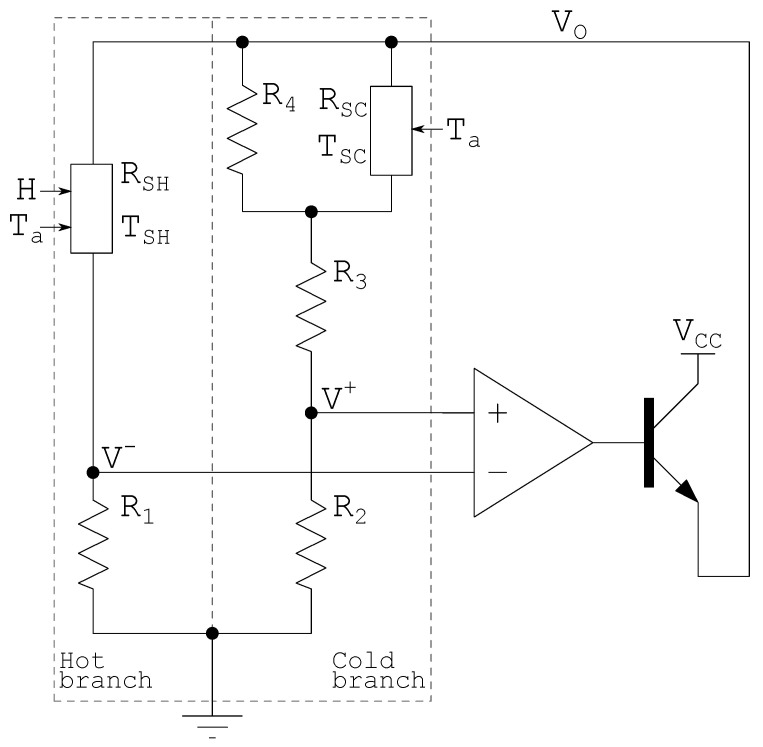
Proposed CTD Wheatstone bridge architecture [13].

The sensor responsible for sensing the ambient temperature is called cold sensor ($R_{sc}$), while the sensor responsible for receiving incident radiation is called hot sensor ($R_{sh}$). The constant ΔT represents the temperature difference between the hot and cold sensors. The branch that contains the hot and cold sensors are referred to as the hot branch and cold branch, respectively. Figure 3 shows the proposed Wheatstone CTD bridge architecture using NTCs.

Like CT architecture, the OpAmp also provides a positive voltage to the transistor base, which is used to increase the current capacity. However, the transistor may be reverse-biased if the heated sensor temperature saturates and/or the OpAmp offset voltage is negative. Both cases can cause the circuit to stop functioning correctly. It is also important that the transistor $V_{cb}$ threshold be greater than the differential power supply to avoid damage.

The problem of saturation can be avoided by keeping the OpAmp offset positive and adding a reference resistor $R_{ce}$ between nodes $V_{CC}$ and $V_{o}$ that provides a minimum current for the OpAmp inputs to function properly. The $R_{ce}$ guarantees a minimum voltage reference so that the OpAmp returns to provide a positive voltage output when the sensor returns to the normal temperature condition.

The hot sensor in the CTD Wheatstone bridge architecture is chosen in a similar manner to the one in the CT Wheatstone bridge architecture. The value of $R_{1}$ is optimized to provide the maximum voltage variation in the Wheatstone-Bridge over the change of $T_{a}$. This guarantees the maximum possible linearization over the ambient temperature range of the nonlinear sensor-based transfer function. The CTD Wheatstone bridge architecture value for ($R_{1}$) is chosen to be the geometric mean of the extreme sensor values. The CTD Wheatstone bridge architecture optimal value for ($R_{1}$) is:

(13) $$R_{1}=\sqrt{R_{sh}\left(\min\left(T_{a}\right)+\Delta T\right)\cdot R_{sh}\left(\max\left(T_{a}\right)+\Delta T\right)},$$

where $R_{sh}\left(\cdot \right)$ is the hot sensor static response.

To keep the hot sensor ($R_{sh}$) heated up ΔT over the ambient temperature is necessary to satisfy the bridge balance that, neglecting the $V_{os}$ effect, is:

(14) $$\frac{R_{1}}{R_{1}+R_{sh}\left(T_{a}+\Delta T\right)}\approx \frac{R_{2}}{R_{2}+R_{3}+\frac{R_{sc}\left(T_{a}\right)\cdot R_{4}}{R_{sc}\left(T_{a}\right)+R_{4}}}.$$

Resistors $R_{3}$ and $R_{4}$ serve to compensate for the intrinsic temperature of the cold sensor. A good choice of $R_{sc}$ presents a high value of $R_{25}$ and β similar to $R_{sh}$. The high value of $R_{25}$ prevents the cold sensor from self-heating.

One methodology to find $R_{2}$, $R_{3}$ and $R_{4}$ values employs a nonlinear system approximated by three or more points of ambient temperature. A good practice is to use the maximum, average and minimum points of ambient temperature. Other constraints of the nonlinear system are the values of $R_{2}$, $R_{3}$ and $R_{4}$ which cannot be negative and the cold branch must have enough resistance to prevent $R_{sc}$ from self-heating. This way, the nonlinear system is given by:

(15) $$\left\{\begin{array}{c} \frac{R_{1}}{R_{1}+R_{sh}\left(\min(T_{a})+\Delta T\right)}=\frac{R_{2}}{R_{2}+R_{3}+\left(\frac{R_{sc}\left(\min(T_{a})\right)\cdot R_{4}}{R_{sc}\left(\min(T_{a})\right)+R_{4}}\right)}\\ \frac{R_{1}}{R_{1}+R_{sh}\left(\bar{T_{a}}+\Delta T\right)}=\frac{R_{2}}{R_{2}+R_{3}+\left(\frac{R_{sc}\left(\bar{T_{a}}\right)\cdot R_{4}}{R_{sc}\left(\bar{T_{a}}\right)+R_{4}}\right)}\\ \frac{R_{1}}{R_{1}+R_{sh}\left(\max(T_{a})+\Delta T\right)}=\frac{R_{2}}{R_{2}+R_{3}+\left(\frac{R_{sc}\left(\max(T_{a})\right)\cdot R_{4}}{R_{sc}\left(\max(T_{a})\right)+R_{4}}\right)}\\ R_{2}>0\\ R_{3}\geq 0\\ R_{4}\geq 0\\ \frac{R_{4}^{2}\cdot V_{o}^{2}\cdot R_{sc}\left(\max\left(T_{a}\right)\right)}{R_{sc}\left(\max\left(T_{a}\right)\right)\cdot \left(R_{2}+R_{3}+R_{4}\right)+R_{4}\cdot \left(R_{2}+R_{3}\right)}⪅G_{th-sc}\cdot \zeta \end{array}\right.,$$

where $G_{th-sc}$ [W/${}^{\circ }$C] are the thermal dissipation factor for hot and cold sensors, respectively, and ζ [${}^{\circ }$C] the maximum allowable self-heat of the cold sensor. The solution of (15) can be achieved using an optimization algorithm like the trust-region or Levenberg–Marquardt.

### 2.5. Experimental Setup

We prototyped the proposed CTD Wheatstone bridge using a glass dome, a metal case, and a printed circuit board for the electronic components. The printed circuit board contains the connectors to input/output and the conditioning circuit that is composed of: 2PC4081R as a bipolar NPN transistor, the TL081 as operational amplifier with offset adjustment, and two NTCs thermistors are employed as hot and cold sensors.

**Figure 4:**
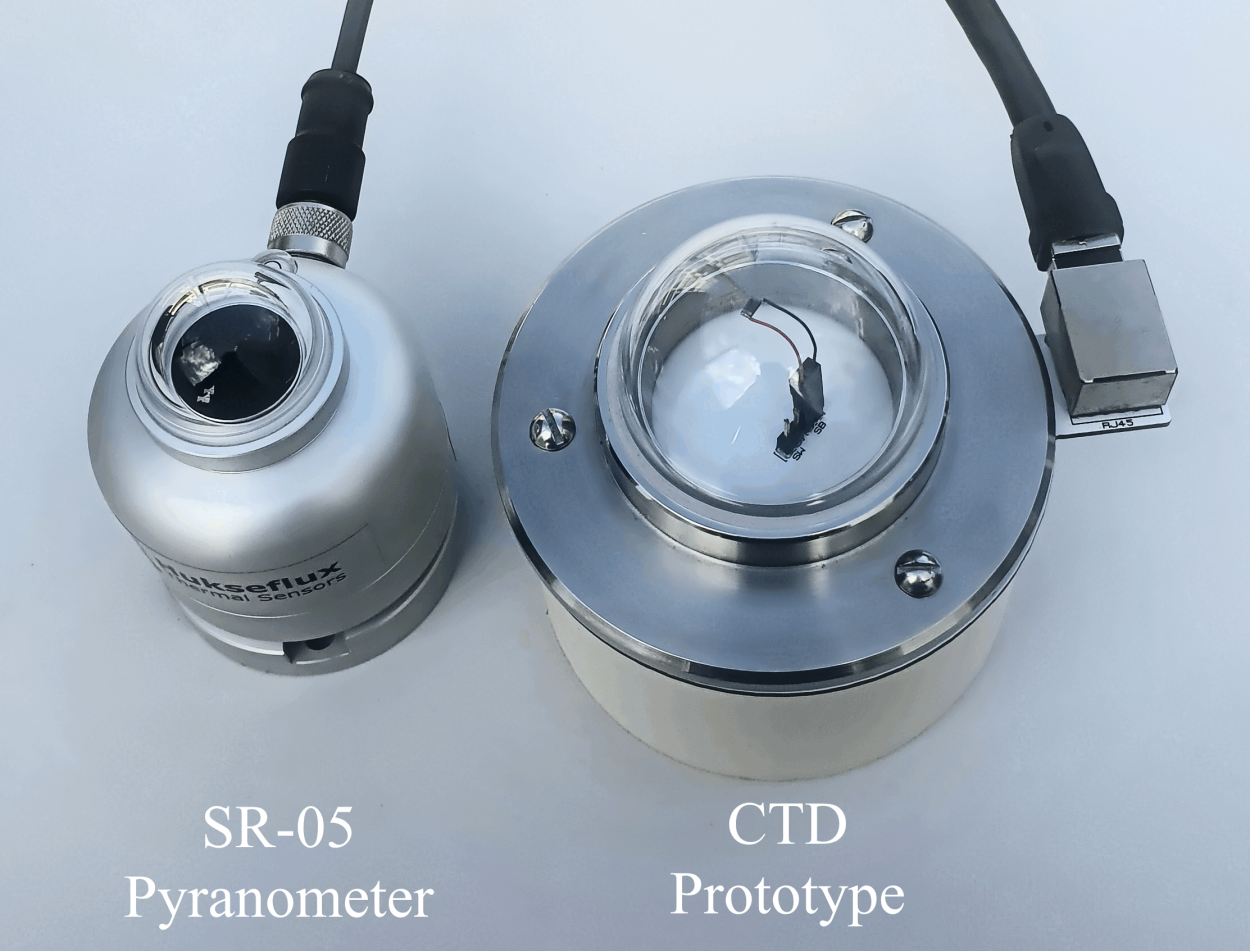
The SR-05 pyranometer and CTD prototype.

Afterwards, to validate our proposed architecture, we compared the experimental results with the Hukseflux SR-05 thermopile pyranometer. Figure 4 shows the commercial SR-05 pyranometer and proposed CTD prototype from a perspective view.

**Figure 5:**
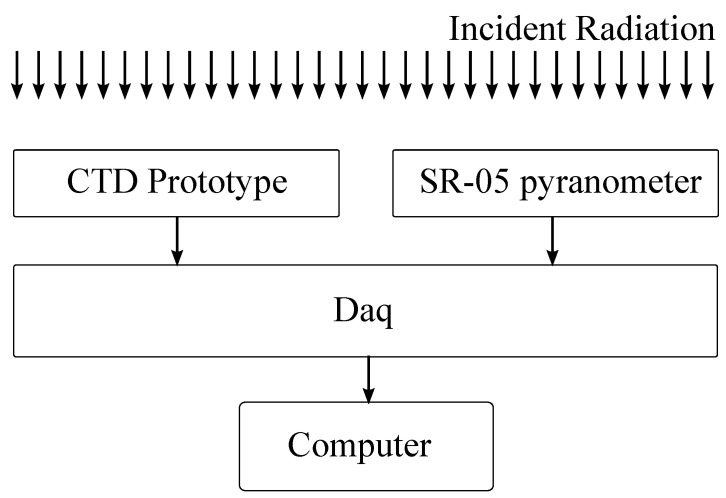
The setup diagram of performed experiments.

The data of each pyranometer were sampled using a Data Acquisition Device (DAQ), and processed in a computer to compute the incident radiation, as illustrated in Figure 5. The DAQ used is a NI USB-6361 and the computer presented the following configuration: AMD A8-5500 @ 3.2 GHz Quad-core 32 nm, 4 GB of RAM, 512 GB HD and a 3.0 USB port. Two 12 volt @ 7 ampere-hour batteries were used as a pyranometer power supply.

The data acquisition device was configured to read the differential mode with a resolution of 16 bits, −10 to 10 V input range and a sampling rate of 100 Sa/s. The SR-05 pyranometer signal was acquired through the 4–20 mA analog output, and the CTD prototype signal acquired by nodes $V_{o}$ and $V^{-}$.

A Fluke 1502A thermometer and a Keysight DMM 34470 were used to calibrate the CTD prototype sensors’ static response. The dynamics response calibration was performed applying a Pseudo-Random Multilevel Sequence (PRMS) in a current source with Daq [30,31,32]. The dynamics response obtained was used in a system identification toolbox to obtain the dynamics parameters of the system [24]. A search method of Levenberg–Marquardt was applied with the current range between 300 μA to 40 mA. Experimental results and CTD parameters obtained are shown in Section 3.

## 3. Results and Analysis

### 3.1. Analysis of CT and CTD Wheatstone Bridge Architecture

To analyze the performance of the CT and CTD Wheatstone bridge architecture, consider that the ambient temperature varies from 0 to 60 ${}^{\circ }$C, and the incident radiation varies between 0 and 1600 kW/m${}^{2}$ and $\max\left(V_{o}\right)=12$ V. The sensor and parameters for the CT and CTD architectures are summarized in Table 2 and Table 3, respectively. The parameters presented were adapted from [1,7,11] with computed values from Equation (12), which avoid any temperature saturation, and (13), which optimize the value of $R_{1}$ for CTD architecture.

Applying the parameters from Table 3 to (15), it is possible to obtain the relation of resistances that allow a constant temperature difference between hot and cold sensors. The resistance values obtained for the parameters used were: $R_{2}=27.3$ kΩ, $R_{3}=514\;\Omega$ and $R_{4}=1$ MΩ. These resistances values allow a fit of 99.6% in the comparison of the transfer functions of the hot branch and the cold branch.

**Figure 6:**
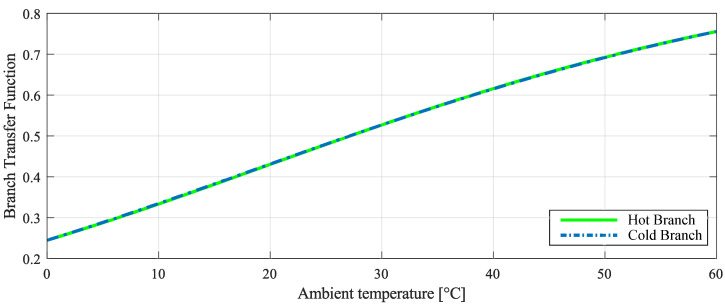
Transfer function of hot and cold branches in the CTD Wheatstone bridge.

Figure 6 shows the hot and cold branch transfer function of CTD Wheatstone bridge architecture. In order for ΔT be constant, it is necessary that hot and cold branches’ transfer functions, represented respectively by $V^{-}/V_{o}$ and $V^{+}/V_{o}$, are the same for the entire ambient temperature range, as described in (). If the hot branch and cold branch transfer functions are not same, it will lead to a ΔT different from the one projected for the heated sensor and, consequently, to an error in the incident radiation estimation.

**Figure 7:**
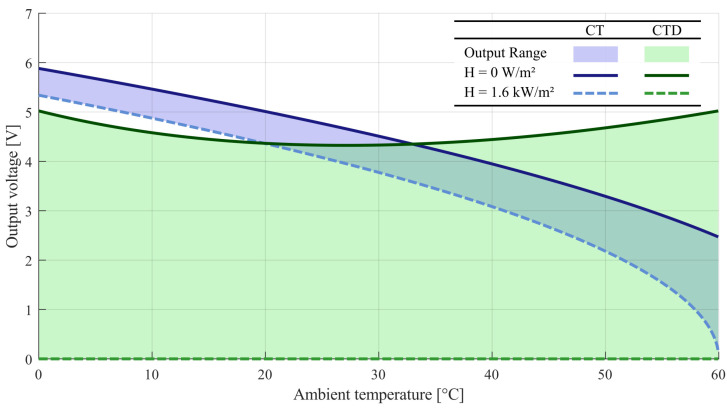
The output voltage response from CT and CTD architecture.

The values from Table 2 and Table 3 were applied in the simulation of CT and CTD Wheatstone bridge architectures. From (9) and (11), the output voltage span was obtained as a function of ambient temperature and incident radiation, as shown in Figure 7.

From Figure 7, it can be observed that the output voltage varies significantly between the architectures. For comparing the performance of both architectures, the metrics of average sensitivity $\bar{S}$, sensitivity variation ΔS, and useful voltage range $\epsilon_{V_{o}}$, defined as:

(16) $$\bar{S}=\frac{\bar{\Delta V_{o}\left(T_{a}\right)}}{\Delta H},$$

(17) $$\Delta S=\frac{\max\left(\Delta V_{o}\left(T_{a}\right)\right)-\min\left(\Delta V_{o}\left(T_{a}\right)\right)}{\Delta H},$$

(18) $$\epsilon_{V_{o}}=\bar{\frac{\Delta V_{o}\left(T_{a}\right)}{\max(V_{o})}}\times 100\%.$$

Ideally, the sensitivity should be as high and constant as possible, while the useful voltage range should be 100%. The following metrics were obtained from results (Table 4):

Compared to the CT architecture, the proposed architecture has a 5-fold increase in average sensitivity, approximately 3-fold decrease in sensitivity variation, and a 76.3% increase in the useful output voltage range.

**Figure 8:**
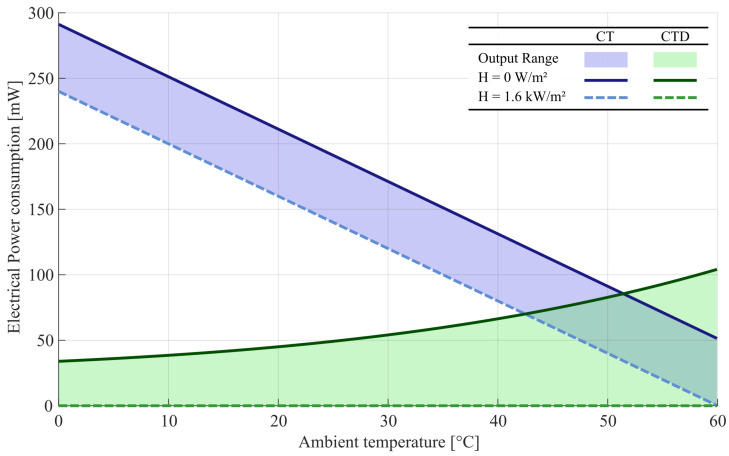
The power consumption from a sense branch in CT and CTD Wheatstone bridge architectures.

Considering that the electrical power is mostly dissipated in the branch that contains the hot-sensor, one can estimate this branch electrical power consumption considering only the heated sensor and the resistor $R_{1}$ [13]. Figure 8 illustrates the hot branch highest electrical power consumption in steady-state by ambient temperature.

Figure 8 shows that CT architecture power consumption is inversely proportional to the ambient temperature while the CTD architecture increases nonlinearly with ambient temperature. In this case, the CT is more efficiently in ambient temperature above 50 ${}^{\circ }$C due to the low value of $R_{1}$.

### 3.2. SPICE Simulation

A simulation was also performed using a SPICE environment with the classic architecture (CT) and the proposed architecture (CTD), considering varying both the ambient temperature and the incident radiation. The objective of this simulation was to verify if the temperature of the heated sensor would follow the intended reference and to explore the effects of using the temperature difference in the estimation of H.

The Wheatstone CT and CTD bridge architecture, shown in Figure 2 and Figure 3, were simulated using a thermistor model provided in [1]. The simulation parameters are summarized as follows: Table 5 contains the CT Wheatstone bridge architecture sensor parameters; Table 6 contains the CT Wheatstone bridge architecture fixed resistances that allow $T_{s}$ to be 72.8${}^{\circ }$C; Table 7 contains the CTD Wheatstone bridge architecture sensor parameters; Table 8 contains the CTD Wheatstone bridge architecture fixed resistances that allow ΔT to be 12.8${}^{\circ }$C; Table 9 contains simulation parameters shared between CT and CTD Wheatstone bridge architecture. The parameters $V_{os}$ represent the offset voltage and $A_{0}$ the open loop gain of the operational amplifier.

The ambient temperature and incident radiation are defined, respectively, in simulation as $T_{a}\left(t\right)=30+30\cdot \sin\left(2\cdot \pi \cdot \left(1/3600\right)\cdot t\right)$ and $H\left(t\right)=1600\cdot \left(u(t-1200)-u(t-2400)\right)$, where u(·) is the step function.

**Figure 9:**
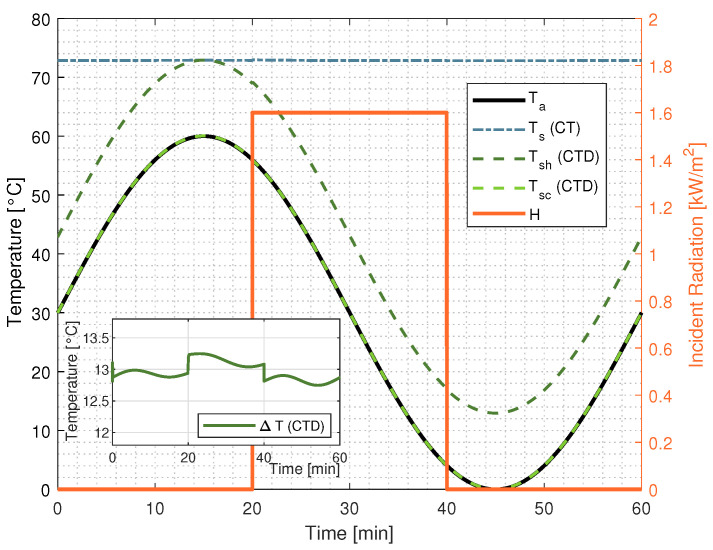
CT and CTD architectures’ sensor temperatures from SPICE simulations.

Figure 9 shows the ambient temperature $T_{a}$, the sensor temperature $T_{s}$ in CT architecture, the hot $T_{sh}$ and cold $T_{sc}$ sensor temperature in CTD architecture, the incident radiation H, and the difference of temperature ΔT.

As evidenced by Figure 9, the sensor temperature $T_{s}$ of the classical CT Wheatstone bridge architecture remains practically constant regardless of the ambient temperature, while the temperature of the hot sensor $T_{sc}$ in the CTD Wheatstone bridge architecture remains ΔT degrees warmer than the ambient temperature.

**Figure 10:**
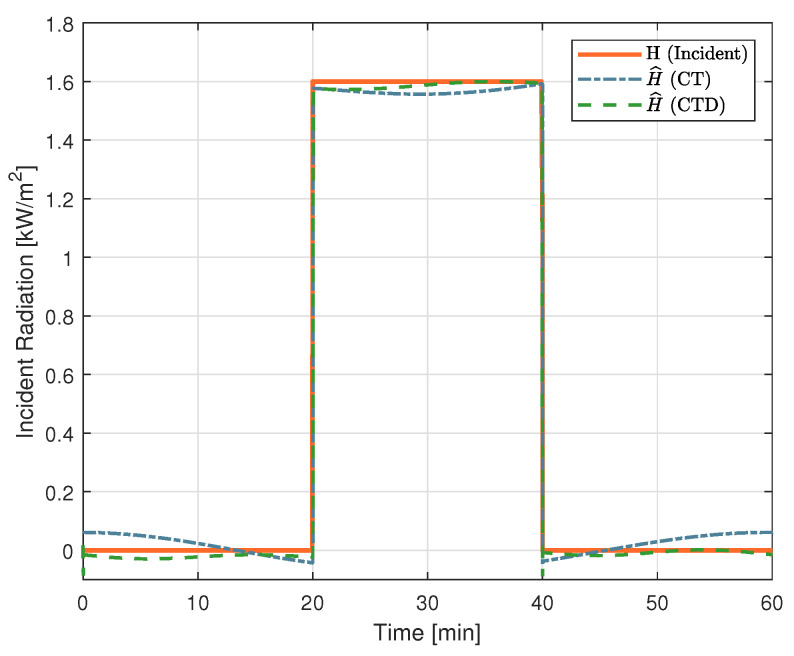
Estimation of Incident Radiation in SPICE simulation.

To estimate the incident radiation, (5) can be used as:

(19) $$\hat{H}=\frac{G_{th}\cdot \left(T_{s}-T_{a}\right)-\left(V_{o}-V^{-}\right)\cdot \frac{V^{-}}{R_{1}}}{\alpha },$$

where $\hat{H}$ [W/m${}^{2}$] is the estimated incident radiation. The estimated incident radiation for CT and CTD Wheastone bridge architectures is shown in Figure 10. The $T_{a}$ sensing in CT architecture simulation followed the cold sensor time constant for fair comparison.

Small fluctuations in sensor temperature (CT) or temperature difference (CTD) lead to a small error that becomes noticeable due to amplification caused by α. The mean absolute error of simulated architectures was 39±45 W/m${}^{2}$ for CT and 6±30 W/m${}^{2}$ for CTD.

### 3.3. Experimental Results

The developed prototype was submitted to a field experiment with the Hukseflux SR-05 pyranometer as a reference. Before the measurement, the sensor calibration was performed as described in Section 2.5. Table 10 presents the static and dynamics sensor parameters used in CTD prototypes.

The pyranometer was exposed to the sun and observed the maximum temperature difference between the hot and cold sensors, without control, in order to find the maximum temperature variation and α parameter, according to (12). The maximum difference observed was ΔT=11.3${}^{\circ }$C, so the project parameter was set to ΔT=12${}^{\circ }$C. From ΔT and the sensors’ static response, a new calculus of the bridge resistances was performed. The values of resistances are summarized in Table 11, and other project parameters are summarized in Table 12.

All measurements were performed for 20 min in three different weather conditions, at the Federal University of Rio Grande do Norte, RN, Brazil, with the following geospatial coordinates: −5${}^{\circ }$50${}^{\prime }$32.6034${}^{\prime \prime }$ (lat.), −35${}^{\circ }$11${}^{\prime }$50.9274${}^{\prime \prime }$ (long.).

The SR-05 pyranometer and the CTD prototype results were acquired by DAQ and digitally processed to estimate incident radiation, the measurement error, and the sensors’ temperature. A low-pass filter, with a cutoff frequency of 10 hertz, was applied to the prototype CTD signal to remove high-frequency components. The filter was not applied to the SR-05 as this device already has a low frequency response.

**Figure 11:**
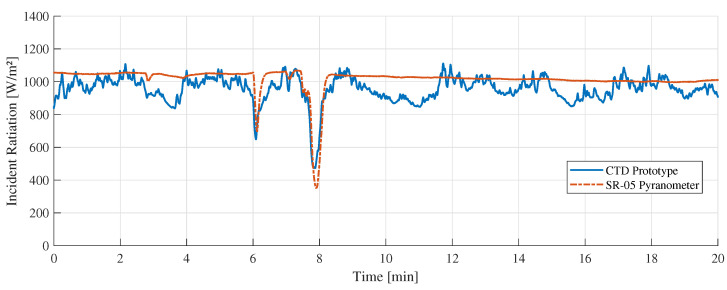
“A” measurement with clear sky weather conditions.

**Figure 12:**
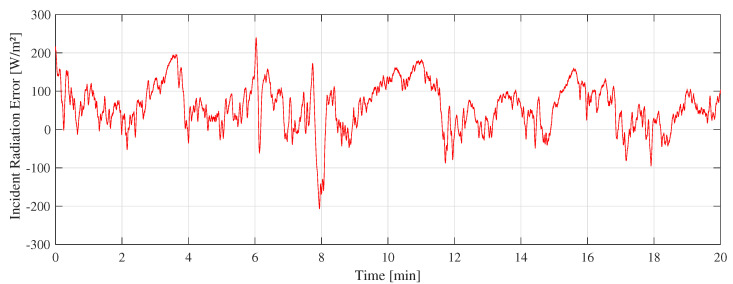
Difference between SR-05 and CTD prototype in A measurement.

**Figure 13:**
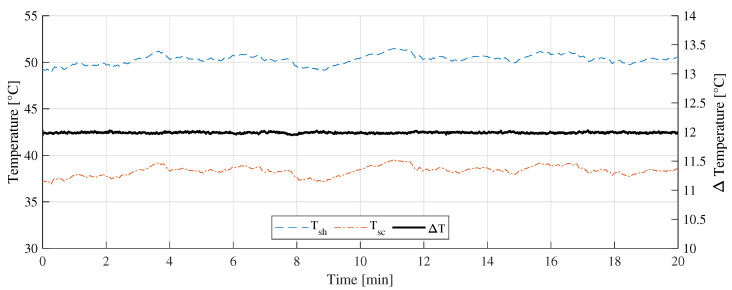
Temperature measurement during the A measurement.

Figure 11 shows the “A” measurement taken on 16 February 2022 at 11:10 a.m. with clear sunny conditions. During the experiments, few cumulus clouds in the sky were noticed. Figure 12 shows the measurement difference between the CTD prototype and the pyranometer in “A” measurement. The absolute error measured was 70±48 W/m${}^{2}$. Figure 13 shows the temperatures of the hot sensor ($T_{sh}$), cold sensor ($T_{sc}$), and the temperature difference (ΔT) during “A” measurement.

**Figure 14:**
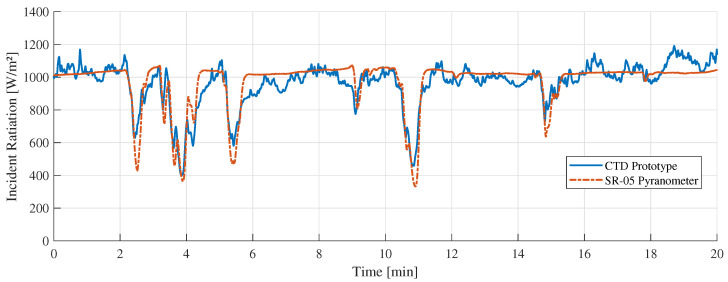
“B” measurement with partially cloudy conditions.

**Figure 15:**
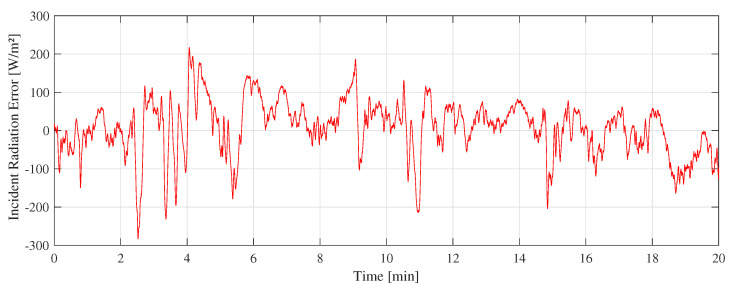
Difference between SR-05 and CTD prototype in B measurement.

**Figure 16:**
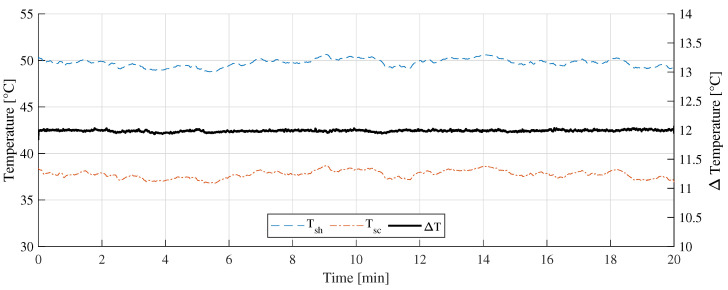
Temperature measurement during the B measurement.

Figure 14 shows the “B” measurement taken on 14 February 2022 at 11:10 a.m. with partially cloudy conditions. During the experiments, cumulus and stratocumulus clouds were present. Figure 15 shows the measurement difference between the CTD prototype and the pyranometer in “B” measurement. The absolute error measured was 55±45 W/m${}^{2}$. Figure 16 shows the temperatures of the hot sensor ($T_{sh}$), cold sensor ($T_{sc}$), and the temperature difference (ΔT) during “B” measurement.

**Figure 17:**
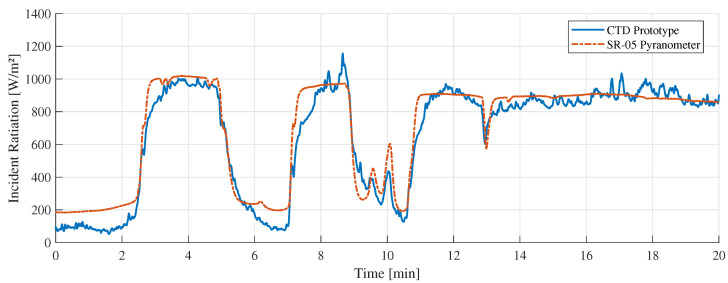
“C” measurement with cloudy weather conditions.

**Figure 18:**
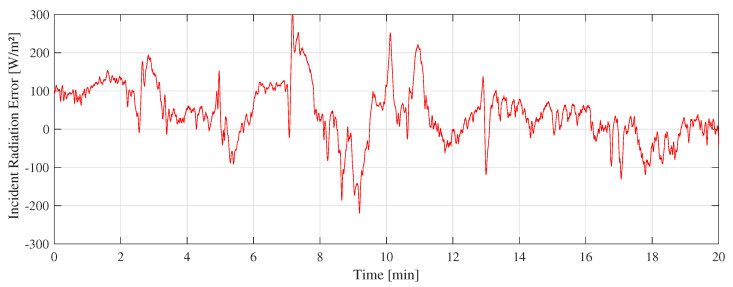
Difference between SR-05 and CTD prototype in C measurement.

**Figure 19:**
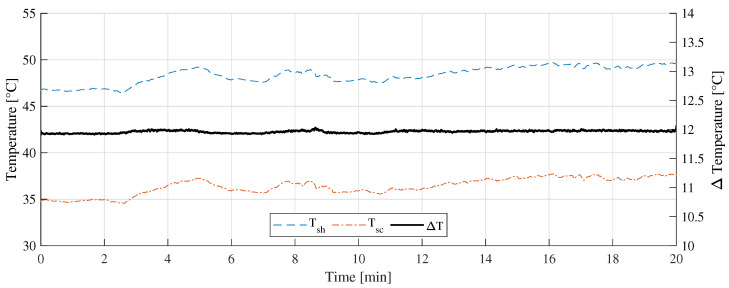
Temperature measurement during the C measurement.

Figure 17 shows the “C” measurement taken on 18 February 2022 at 11:30 a.m. with cloudy weather conditions. Nimbostratus clouds were present but without rain. Figure 18 shows the measurement difference between the CTD prototype and the pyranometer in “C” measurement. The absolute error measured was 64±53 W/m${}^{2}$. Figure 19 shows the temperatures of the hot sensor ($T_{sh}$), cold sensor ($T_{sc}$), and the temperature difference (ΔT).

### 3.4. Enhancement and Post-Processing Information

The enhancements can be performed in two ways: (i) in calibration process; (ii) in post-processing. In the calibration process, several measurements can be made simultaneously between calibrated pyranometers and the CTD prototype. The optimization algorithms can be employed to determine the best constant values, $G_{th}$, and α that reduce the measurement difference between the calibrated device and the prototype. This method must be used carefully, and it is recommended only to refine the sensor parameters.

Post-processing methods include smoothing the response using digital filters and applying correction factors. In general, the correction factor is only used if the incident radiation is constructed using only the $V_{o}$ signal. Using signal node $V_{o}$ with signal node $V^{-}$ provides greater reliability in signal acquisition. The voltage signal at nodes $V^{-}$ and $V^{+}$ can be used to determine if the sensor has already reached its steady state, avoiding measurement errors.

From $V_{o}$, $V^{-}$, and $V^{+}$, it is possible to select the best values to reconstruct *H* that happens when $V^{-}$ is equal to $V^{+}$. This prevents any dynamic interference caused by the hot and cold sensor constants. For this, a comparator can be added to nodes $V^{-}$ and $V^{+}$ to generate a trigger signal for the sampling device. The comparator output signal must be one if $V^{-}=V^{+}$ and zero if $V^{-}\neq V^{+}$. Table 13 shows the absolute error using only $V_{o}$, using $V_{o}$ and $V^{-}$, and using all nodes to reconstruct H.

## 4. Discussion

This paper presented the constant temperature difference Wheatstone bridge architecture and shows its analytical model, a SPICE simulation, and a set of field experiments carried out with a developed prototype. Information and methodologies to find the ideal temperature difference for proposed architecture are also provided.

The proposed architecture is intended to mitigate the effects of ambient temperature variation on incident radiation measurement, while maintaining the advantage of fast response of the Wheatstone bridge based architecture. The constant temperature difference use abruptly changes the output voltage range of the circuit, promoting a greater range of useful voltage, with greater constancy without DC level as shown in Figure 7. It is a great advantage especially if it is planned to add an analog-to-digital converter.

The increase of useful output range reflects directly on the device sensitivity that presents more constancy in all ambient temperature range, and in power consumption. The power consumption of our proposed architecture is significantly at lower temperatures when compared with classical architecture, as shown in Figure 8. The energy consumption of the proposed architecture is only higher at high temperatures due to the $R_{1}$ that is generally great in CTD than CT architecture. The power consumption is an important parameter for this type of device as it can be powered by batteries and deployed in remote locations.

A simulation was performed and showed that CTD Wheatstone bridge architecture can increase or decrease the delivered power to the sensor in order to keep it ΔT higher than ambient temperature. An equation was presented to find out the optimized ΔT value. From the parameters, it is possible to estimate the incident radiation simulated. Simulated values were compared and showed that our proposal provides lower uncertainty than classical architecture. This happens because there is a greater margin for temperature variation compensation by OpAmp when using the difference instead constant temperature.

The simulation results corroborate the theory by keeping the difference between the sensors almost constant. There is also a small fluctuation that occurs in the sensor temperature due to the sensors thermal capabilities ($C_{th}$). Usually this variation is minimal but eventually becomes noticeable due to amplification by the calibration constant (α). This effect can be reduced by considering the actual temperature instead of the constant in the reconstruction equation (19).

A set of field experiments were performed with the proposed CTD pyranometer that is compared to SR-05 as a reference pyranometer. The main difference between the SR-05 pyranometer and proposed CTD pyranometer is the response time. The SR-05 has a response time of ≈18 s, while our proposed architecture shows a response time in milliseconds due to the high proportional feedback gain.

The CTD pyranometer variation is directly associated with its dynamics constants. While the temperature presents high precision, due to the high gain in closed-loop, the incident radiation is amplified in an open loop by the low value of α constant. In order to smooth the response and reduce the uncertainty, it is desirable to increase the α constant and reduce the dissipation factor $G_{th}$. This may allow for increasing the temperature difference and, consequently, reducing the variation.

One way to reduce the variation in the proposed prototype response is adding a low-pass filter to the OpAmp output voltage or by increasing the α calibration constant. The α calibration constant can be improved by using a thin layer of special inks with high light absorption capacity.

Figure 11, Figure 14 and Figure 17 showed a good similarity between the CTD prototype and the SR-05 pyranometer in different weather conditions. In some specific points, there is a noticeable variation only in our prototype. This contrast is explained by the different spectral device responses, which can be adjusted by covering the sensor with the same special black ink used in the commercial pyranometer. Despite this, all valleys and peaks are perceived to a similar degree and intensity in both devices.

The difference between the CTD prototype and the SR-05 pyranometer shown in Figure 12, Figure 15, and Figure 18 is similar to a random variable with a non-zero mean. The main challenge is to keep its dispersion as low as possible with an average close to zero. To reach this objective, this error can be employed in minimization algorithms to improve calibration of CTD pyranometers.

Regarding the temperature differences shown in Figure 13, Figure 16, and Figure 19, it can be concluded that, as the incident radiation increases, the ambient temperature also increases. In all the cases presented, the controller managed to keep the temperature difference stabilized at 12 ${}^{\circ }$C and thus drastically reduce the influence of the ambient temperature variation on the measurement.

The main contributions of this work were to analyze classical architecture from a different perspective, proposing to use the temperature difference instead of the constant temperature. A method was developed to find parameters that allow the Wheatstone bridge to keep the sensor warm to ΔT degrees above ambient temperature and to carry out simulations and field experiments to validate the proposed architecture.

In this work, the fundamentals were applied to Wheatstone bridge architecture, but can be adapted to other architectures, analog or digital. This method can also be adapted to a Wheatstone bridge anemometer.

## 5. Conclusions

In this work, a modification of the constant temperature architecture for constant temperature difference in the Wheatstone bridge was presented. This modification allows for mitigating the interfering effects of ambient temperature variation in the measurement of solar radiation. Parameters such as circuit output range, sensitivity, and energy consumption were investigated, which respectively showed a greater constancy in the voltage range, an increase of almost 5 times in sensitivity and lower energy consumption. To validate the analysis, SPICE simulations were performed, which showed a reduction in measurement uncertainty due to ambient temperature variation. In this work, a prototype of the proposed architecture was also developed, which was compared with a commercial pyranometer. Experimental results show high similarity between the response of the developed prototype and the commercial pyranometer with an average absolute error of 63 W/m${}^{2}$ and maximum uncertainty of 53 W/m${}^{2}$.

## 6. Patents

This research resulted in two patents deposited at the National Institute of Intellectual Property (INPI) of Brazil, as a utility model, by the Innovation Agency of the University of Rio Grande do Norte. The date field follows ISO 8601.
Identifier:   **BR 20 2022 012953 9**Status:Deposited in INPI (Brazil)Date:2022/07/21Title [en]:Electrical Equivalence Radiometer with Constant Temperature DifferenceTitle [pt]:Radiômetro de Equivalência Elétrica com Diferença de Temperatura ConstanteInventors:E.C.S.D., J.T.D.S. and S.Y.C.C.
Identifier:   **BR 20 2022 014425 2**Status:Deposited in INPI (Brazil)Date:2022/06/29Title: [en]Current Mirror Architecture for Electrical Equivalence Radiometers and
AnemometersTitle: [pt]Arquitetura de Espelho de Corrente para Radiômetros e Anemômetros de
Equivalência ElétricaInventors:E.C.S.D., J.T.D.S. and S.Y.C.C.

## Figures and Tables

**Table 1:** State of the Art summarized in the last 6 years.

Ref.	Year	Description
[12]	2022	Presented a differential and constant temperature current based architecture using NTC sensors to measure incident radiation.
[13]	2022	Presented a differential constant temperature Wheatstone-bridge architecture using NTC sensors for measure incident radiation.
[14]	2022	Presented analysis about variance propagation in closed-loop systems using negative temperature coefficient thermistors. The analysis was performed over ambient temperature and the equilibrium temperature between sensor and the environment.
[1]	2021	Presented the influence of ambient temperature variation on classical Wheatstone bridge architecture output voltage and sensitivity. This paper also presented the $V_{os}$ effect on sensor settling time.
[15]	2020	Proposed an estimation method to compensate for the error caused in the measurement during the transient regime in thermistor-based systems operating in the constant temperature configuration.
[7]	2019	Proposed an autorange method for adjusting the measurement range of the thermal sigma-delta converter. The applied method was used to measure solar radiation.
[16]	2017	Presented the analytical performance for Wheatstone-bridge Constant Temperature architecture using an NTC for fluid speed measurement. Contributed with several parameters such as: settling time, sensitivity and consumption.
[11]	2016	Presented the thermal-sigma delta converter for incident radiation measurement. Some highlights of the work were the noise analysis and the consumption ratio.

**Table 2:** CT parameters used in simulation.

Parameter	Value	Parameter	Value
*H*	0 to 1600 W/m${}^{2}$	$T_{a}$	0 to 60 ${}^{\circ }$C
$G_{th}$	2 mW/${}^{\circ }$C	α	16 μm${}^{2}$
$R_{s}\left(25\right)$	330 Ω	β	3700 K
$T_{s}$	72.8 ${}^{\circ }$C	$R_{1}$	59.4 Ω

**Table 3:** CTD parameters used in simulation.

Parameter	Value	Parameter	Value
*H*	0 to 1600 W/m${}^{2}$	$T_{a}$	0 to 60 ${}^{\circ }$C
$G_{th}$	2 mW/${}^{\circ }$C	α	16 μm${}^{2}$
$R_{sh}\left(25\right)$	330 Ω	$\beta_{sh}$	3700 K
$R_{sc}\left(25\right)$	30 kΩ	$\beta_{sc}$	3600 K
ΔT	12.8 ${}^{\circ }$C	$R_{1}$	182.5 Ω

**Table 4:** CT and CTD metrics.

Metric	CT	CTD
$\bar{S}$ (V· m${}^{2}$/W)	$0.5\times 10^{-3}$	$2.9\times 10^{-3}$
ΔS (V· m${}^{2}$/W)	$1110\times 10^{-6}$	$435\times 10^{-6}$
$\epsilon_{V_{o}}$ (%)	14.4	90.7

**Table 5:** CT Wheatstone bridge architecture sensor parameters used in SPICE simulation.

Sensor	$R_{25}\;\left[\Omega \right]$	β [K]	$G_{\mathrm{th}}$ [W/${}^{\circ }$C]	$C_{\mathrm{th}}$ [J/${}^{\circ }$C]	α [m${}^{2}$]
Hot Sensor	330	3700	$2\times 10^{-3}$	$10\times 10^{-3}$	$16\times 10^{-6}$

**Table 6:** CT Wheatstone bridge architecture fixed resistors used in SPICE simulation.

Resistor	Value
$R_{1}$	59.4Ω
$R_{2}$	47 kΩ
$R_{3}$	47 kΩ

**Table 7:** CTD Wheatstone bridge architecture sensor parameters used in SPICE simulation.

Sensor	$R_{25}\;\left[\Omega \right]$	β [K]	$G_{\mathrm{th}}$ [W/${}^{\circ }$C]	$C_{\mathrm{th}}$ [J/${}^{\circ }$C]	α [m${}^{2}$]
Hot Sensor	330	3700	$2\times 10^{-3}$	$10\times 10^{-3}$	$16\times 10^{-6}$
Cold Sensor	$30\times 10^{3}$	3600	$4\times 10^{-3}$	$20\times 10^{-3}$	-

**Table 8:** CTD Wheatstone bridge architecture fixed resistors used in SPICE simulation.

Resistor	Value
$R_{1}$	182.5Ω
$R_{2}$	27.3 kΩ
$R_{3}$	514Ω
$R_{4}$	1 MΩ

**Table 9:** Common parameters used in SPICE simulation.

Parameter	Value
$V_{os}$	2 mV
$A_{o}$	$10^{6}$
$V_{cc}$	12 V
$V_{ee}$	−12 V

**Table 10:** Experimental CTD Prototype sensor parameters.

Sensor	$R_{25}\;\left[\Omega \right]$	β [K]	$G_{\mathrm{th}}$ [W/${}^{\circ }$C]	$C_{\mathrm{th}}$ [J/${}^{\circ }$C]	α [m${}^{2}$]
Hot Sensor	314.7	3864	$2.46\times 10^{-3}$	$8.58\times 10^{-3}$	$185\times 10^{-7}$
Cold Sensor	$29.3\times 10^{3}$	3633	$3.01\times 10^{-3}$	$12.68\times 10^{-3}$	-

**Table 11:** Wheatstone bridge fixed resistors in the CTD Prototype.

Resistor	Value
$R_{1}$	175Ω
$R_{2}$	26.7 kΩ
$R_{3}$	96Ω
$R_{4}$	2.7 MΩ

**Table 12:** Other parameters in the CTD Prototype.

Parameter	Value
$V_{os}$	2 mV
$V_{cc}$	12 V
$V_{ee}$	−12 V
ΔT	12${}^{\circ }$C

**Table 13:** Absolute error from H reconstruction by different nodes usage.

Absolute Error Using	“A” Measurement	“B” Measurement	“C” Measurement
$V_{o}$ node	73±50 W/m${}^{2}$	57±47 W/m${}^{2}$	64±52 W/m${}^{2}$
$V_{o}$ and $V^{-}$ nodes	70±48 W/m${}^{2}$	55±45 W/m${}^{2}$	64±53 W/m${}^{2}$
$V_{o}$, $V^{-}$ and $V^{+}$ nodes	57±42 W/m${}^{2}$	56±46 W/m${}^{2}$	60±51 W/m${}^{2}$

## Data Availability

All data necessary to reproduce this paper are in the text itself. Additional experimental data can be requested from authors via email.
